# The Potential Role of Major Argan Oil Compounds as Nrf2 Regulators and Their Antioxidant Effects

**DOI:** 10.3390/antiox13030344

**Published:** 2024-03-13

**Authors:** Riad El Kebbaj, Habiba Bouchab, Mounia Tahri-Joutey, Soufiane Rabbaa, Youness Limami, Boubker Nasser, Melford C. Egbujor, Paolo Tucci, Pierre Andreoletti, Luciano Saso, Mustapha Cherkaoui-Malki

**Affiliations:** 1 Laboratory of Health Sciences and Technologies, Higher Institute of Health Sciences, Hassan First University, Settat 26000, Morocco; habibabouchab78@gmail.com (H.B.); rabbaasoufian@gmail.com (S.R.); youness.limami@gmail.com (Y.L.); 2 Institut Supérieur des Professions Infirmières et Techniques de Santé, Errachidia 52000, Morocco; 3 Laboratory of Biochemistry, Neuroscience, Natural Resources and Environment, Faculty of Science and Technology, Hassan First University, Settat 26000, Morocco; 4 Centre des Sciences du Goût et de l’Alimentation, CNRS, INRAE, Institut Agro, Université de Bourgogne, F-21000 Dijon, Francemustapha.cherkaoui-malki@u-bourgogne.fr (M.C.-M.); 5 Department of Chemistry, Federal University Otuoke, Otuoke 562103, Bayelsa State, Nigeria; 6 Department of Clinical and Experimental Medicine, University of Foggia, 71122 Foggia, Italy; 7 Department of Physiology and Pharmacology “Vittorio Erspamer”, Sapienza University of Rome, 00185 Rome, Italy

**Keywords:** argan oil, Nrf2, tocopherols, polyphenols, fatty acids

## Abstract

In recent years, research on the discovery of natural compounds with potent antioxidant properties has resulted in growing interest in these compounds due to their potential therapeutic applications in oxidative-stress-related diseases. Argan oil, derived from the kernels of a native tree from Morocco, *Argania spinosa*, is renowned for its rich composition of bioactive compounds, prominently tocopherols, polyphenols, and fatty acids. Interestingly, a large body of data has shown that several components of argan oil activate the nuclear factor erythroid 2-related factor 2 (Nrf2) pathway, playing a crucial role in the cellular defense against oxidative stress. Activation of this Nrf2 pathway by argan oil components leads to the increased expression of downstream target proteins like NAD(P)H quinone oxidoreductase (NQO1), superoxide dismutase (SOD), heme oxygenase 1 (HO-1), and catalase (CAT). Such Nrf2 activation accounts for several health benefits related to antioxidant defense, anti-inflammatory effects, cardiovascular health, and neuroprotection in organisms. Furthermore, the synergistic action of the bioactive compounds in argan oil enhances the Nrf2 pathway. Accordingly, the modulation of the Kelch-like ECH associated protein 1 (Keap1)/Nrf2 signaling pathway by these components highlights the potential of argan oil in protecting cells from oxidative stress and underlines its relevance in dietetic prevention and therapeutic applications. This review aims to provide an overview of how major compounds in argan oil activate the Nrf2 pathway, updating our knowledge on their mechanisms of action and associated health benefits.

## 1. Introduction

Our body naturally generates antioxidants to combat the harmful effects of free radicals. However, an imbalance can result in excess reactive oxygen species (ROS) production, leading to oxidative stress [1]. ROS can be generated by the internal metabolism or after external exposure [2,3]. The excessive production of free radicals can lead to various disorders, including cardiovascular diseases, cancer, and inflammatory pathologies [4]. To combat this oxidative stress, organisms developed a complex system of antioxidants, which can be divided into enzymatic, such as superoxide dismutase (SOD), glutathione peroxidase (GPx), and catalase (CAT), and non-enzymatic, such as glutathione (GSH), ascorbic acid, and thioredoxin [5]. Nuclear factor erythroid 2-related factor 2 (Nrf2) is the main redox-sensitive transcription factor that plays a crucial role in regulating gene expression for molecules that have antioxidant properties [6]. In recent decades, there has been growing interest in bioactive compounds that have a range of biological activities, including anti-inflammatory, anticancer, immunomodulatory, and antioxidant effects [7,8]. In this context, argan oil has gained significant recognition for its unique composition and its numerous health benefits. It is obtained from Argania Spinosa, an endemic tree in the middle of Morocco [9]. Morocco produces 3000 to 4000 tons of argan oil annually, making it the world’s leading producer [10]. Argan oil has traditionally been used for culinary, medicinal, and cosmetic purposes. It is used for joint issues, skin, hair, and nails [11]. Our research team has focused on exploring the antioxidative effect of argan oil and some of its components [12,13,14,15,16,17,18]. The present work first provides a general review of the chemical composition of argan oil compared to other oils. Then, it delves into the antioxidant properties of argan oil as well as its effects on lipid metabolism. Moreover, it describes on the main redox-sensitive transcription factor, Nrf2, and its downstream signaling pathways. Finally, it focuses on the action mechanisms of argan oil and its components on the Nrf2 pathway, aiming to elucidate the antioxidative potential of compounds related to argan oil.

## 2. Composition of Argan Oil

Argan oil has a unique composition marked by elevated levels of linoleic and oleic acids. Moreover, it is rich in polyphenols and tocopherols, which confer antioxidant properties. Additionally, argan oil contains other minor compounds such as carotenoids, squalene, sterols, and xanthophylls. These compounds potentially contribute to the nutritional value of argan oil, its health benefits, its organoleptic characteristics, and its shelf-life [19,20,21].

### 2.1. Glyceride Saponifiable Fraction

The glyceride fraction represents 99% of argan oil, with the majority (95%) being triglycerides [22]. In contrast to other edible oils, an argan oil fatty acid analysis revealed a predominance of oleic acid and linoleic acid, about 80%, showing a balanced amount between monounsaturated and polyunsaturated fatty acids [23]. The proportion of oleic acid in argan oil (43–49%) is higher than that of the oil of sunflowers, soybeans, maize, grape seed, and sesame, whereas this content is lower than that of the oil of olives, almonds, and peanuts (Table 1). Unlike the level of linoleic acid in argan oil, which ranges from 29 to 36%, this concentration is lower than that of the oil of sunflowers, soybeans, maize, grape seed, and sesame, and higher than that of the oil of olives, almonds, and peanuts. Additionally, argan oil is abundant in saturated fatty acids relative to other natural oils and contains a small amount of linolenic acid.

The table below displays a comparison of the main fatty acid compositions between argan oil and other natural oils. The values represent percentages of fatty acids in the total triglyceride fraction.

### 2.2. Unsaponifiable Fraction

The unsaponifiable fraction represents 1% of argan oil and is characterized by a rich composition of sterols and antioxidants such as polyphenols, particularly tocopherols [22]. The composition of the unsaponifiable fraction is mainly influenced by the geographical origin of the argan tree and the process of extraction of the argan oil [31].

#### 2.2.1. Polyphenols

The phenolic composition of argan oil (Table 2) is characterized by the presence of four polyphenols, vanillic acid, syringic acid, ferulic acid, and tyrosol, with a predominance of ferulic acid, which represents more than 94% of the polyphenol fraction [32]. Nonetheless, the polyphenol content of argan oil (3263 μg/kg) is lower compared to olive oil (793 mg/kg); however, it exceeds that of other edible vegetable oils [33]. Polyphenols present in the oils are bioactive molecules that have antioxidant activity. They are primarily responsible for the prevention of auto-oxidation of unsaturated fatty acids, which increases the shelf life of these oils [34]. The pharmacological properties of argan oil are generally attributed to its phenolic compounds.

The table below displays a comparison of the main polyphenol compositions of argan oil and other natural oils. 

#### 2.2.2. Sterols

Table 3 lists the five sterols found in argan oil. The two main sterols are schottenol and spinasterol, while stigmatasol, stigma-8,22-dien-3-ol, and stigma-7,24-dien-3-ol are found in trace amounts [31]. Both spinasterol and schottenol are absent from sunflower and olive oils [20]. Based on previous studies, the sterol content of argan oil (Table 3) remains unaffected by different extraction methods and fruit origins. However, seasonal and regional variations cannot be excluded due to their influences related to the climate and the soil characteristics, respectively [20].

#### 2.2.3. Tocopherols

As natural antioxidants, four isoforms of tocopherols are found in vegetable oils: α-tocopherol (vitamin E), β-tocopherol, γ-tocopherol, and δ-tocopherol [37]. Argan oil contains double the amount of tocopherol as olive oil, with γ-tocopherol constituting the majority at over 75% of the total tocopherols [9]. 

**Table 3:** Chemical composition of argan oil sterols and tocopherols by different extraction methods and in different geographical origins.

Extraction Method	Extraction by Mechanical Press										Artisanal Extraction				Extraction by Organic Solvents		
Roasting	Roasted Argan Seeds					Non-Roasted Argan Seeds					Roasted Argan Seeds			Non-Roasted Argan Seeds			Roasted Argan Seeds
Depuling	Mechanical										Manual	Animal (Goat Dejections )			Mechanical		
**References**	[38]	[39]	[31]	[40]	[20]	[41]	[31]	[31]	[31]	[31]	[41]	[41]	[40]	[40]	[40]	[40]	[40]
**Geographical origin**	Agadir	Taroudant	Tizdi Essaouira	Benaiznassen Chtouka ait baha	Tamanar Essaouira	Tiout Taroudant	Beniznassen Oujda	Ait mzal Chtouka ait baha	Ighrem Taroudant	Tizdi Essaouira	Tiout Taroudant	Tiout Taroudant	Tamanar Essaouira	Tamanar Essaouira	Tamanar Essaouira	Ighrem	Tamanar Essaouira
	**Tocopherols mg/kg of oil**																
**α-Tocopherol**	44.5 ± 6.2	42.23 ± 2.52	26.6	37.2	35	16 ± 1.6	37.2	33.2	49.3	32.7	13 ± 1.3	30 ± 3.0	29.6	33.0	32.0	49.3	29.6
**β-Tocopherol**	3.1 ± 0.8	3.07 ± 0.40													1.1		1.2
**γ-Tocopherol**	616.9 ± 15.8	715.42 ± 7.8	631.3	701.1	480	382 ± 38.2	701.1	615.6	545.9	621.1	345 ± 34.5	283 ± 28.3	619.1	599.3	640.0	545.9	581.3
**δ-Tocopherol**	50.8 ± 6.8	103.22 ± 4.19	59.5	37.2	122	21 ± 2.1	37.2	38.0	38.7	50.9	32 ± 3.2	21 ± 2.1	50.2	46.4	45.4	38.7	56.3
	**Sterol mg/100 g of oil**																
**Schottenol**			46.66	48.47	142	44 ± 2.2	48.47	44.99	46.12	43.39	46 ± 2.3	45 ± 2.3	46.03	47.43	44.62	46.12	45.39
**Spinasterol**			37.07	35.44	115	39 ± 2.0	35.44	39.17	39.29	38.50	38 ± 1.9	42 ± 2.1	36.11	38.54	37.05	39.29	36.91
**Stigmasta-8,22-dien-3-ol**			4.31	4.85	9	5 ± 0.3	4.85	4.77	5.40	4.57	5 ± 0.3	4 ± 0.2	4.08	3.01	4.21	5.40	4.99
**Stigma 7,24--dien-3-ol**			4.81	2.57		6 ± 0.3	2.57	4.71	3.55	5.94	5 ± 0.3	5 ± 0.3	4.48	4.67	6.89	3.55	4.48
**Campesterol**			0.20	0.11			0.11	0.24	0.31	0.17			0.16	0.14	0.16	0.31	0.20

Reported data reveal that the tocopherol content of argan oil can vary significantly between extraction methods and even between fruits grown in the same region (Table 3).

The primary goal of Table 3 is to emphasize the differences in the chemical composition (sterols and tocopherols) of argan oil based on its geographical origin and the extraction method used.

### 2.3. Antioxidant Properties of Argan Oil

The unsaponifiable fraction components have demonstrated antioxidative properties [42]. In mice, following bacterial lipopolysaccharides (LPS) administration, argan oil was able to restore the level of peroxisomal antioxidant enzyme activities, demonstrating its hepatoprotective effect. Accordingly, in both the liver and brain, the LPS-dependent increase in catalase activity, as well as in glutathione peroxidase (GPx) and superoxide dismutase (SOD) activities, can be successfully abrogated by argan oil treatment [14,15]. The LPS-dependent non-enzymatic formation of glutathione in the liver and brain was abolished by argan oil supply. Argan oil is also able to reduce the lipid peroxidation level through malondialdehyde (MDA) measurement during brain and liver injury [15]. Furthermore, a recent paper reported the antioxidant potential of argan oil in mice against iron-inducing oxidative stress in the liver, kidney, and brain. A similar effect was observed in the cultured protozoan *Tetrahymena pyriformis* [13]. Furthermore, exposure to argan oil modulates the peroxisomal antioxidant capacity by upregulating catalase and superoxide dismutase expressions at the translational and post-translational levels [15]. These findings underscore the protective role of argan oil against oxidative stress.

### 2.4. Effects on Lipid Metabolism

Fatty acid synthesis produces oils and fats, which are a source of energy [43]. They are essential to an organism’s growth and development and especially in the regulation of molecular signaling [44].

Indeed, altered levels and the metabolism of fatty acids can contribute to various health concerns in humans, including insulin resistance, obesity, hyperlipidemia, and cardiovascular disease. Therefore, it is critical that the body regulate its fatty acid levels [45]. This metabolic adaptation is performed mainly in the liver by the activation of the nuclear receptor peroxisome proliferator-activate receptor (PPAR)-α. This PPAR isotype is predominantly expressed in several tissues, such as the liver, heart, kidneys, brain, spleen, intestine, and stomach. PPARα target genes are related to fat metabolism and lipid transport and play an important role in fatty acid oxidation [46]. The ligand-dependent activation of this PPARα [47] leads to the recruitment of the coactivator peroxisome proliferator-activate receptor γ-Coactivator (PGC)-1α and then permits the regulation of the transcription of genes encoding peroxisomal and mitochondrial enzymes involved in the hepatic pathways of fatty acid β-oxidation (FAOx) [48,49]. Accordingly, it has been shown that argan oil has a protective effect against the decreased expression of genes involved in hepatic gluconeogenesis and FAOx. This effect might be associated with the recovery of gene expression of the nuclear receptor PPARα and its coactivator PCG-1α [50]. 

Among the classes of fatty acids identified as ligands for PPARs, unsaturated long-chain fatty acids stand out, constituting a significant part, exceeding 80% in both argan oil and olive oil. Indeed, these natural oils are rich in a monounsaturated oleic acid, which represents 46% and 76% of the total composition of argan oil and olive oil, respectively [23]. 

Fundamental studies have shown that oleic acid has a beneficial impact on the cardiovascular risk and lipid profile [51]. Administration of this fatty acid to humans decreases the concentration of LDL and TG [52]. Hence, the substitution of carbohydrates and saturated fat with oleic acid led to a reduction in blood sugar and blood pressure accompanied by an increase in HDL in diabetic patients [53]. These variations in the triglyceride levels are attributed to the increased oxidation of fatty acids through the induction of β-oxidation, a result of oleic acid’s activation of PPARα and the reduction in sterol regulatory element-binding protein (SREBP) activity, thereby decreasing lipogenesis. Moreover, this dietary fatty acid activates PPARα and PPARγ to promote fat oxidation and reduce insulin resistance, which consequently leads to a reduction in hepatic steatosis [54].

Fatty acid intake can effectively modulate liver lipid metabolism [51]. Thus, treatment of mice with olive oil leads to the accumulation of hepatic triglycerides, partly due to increased lipogenesis enzyme activity [55]. Conversely, inhibiting carnitine palmitoyl transferase I (CPT-I) [56] and carnitine/acylcarnitine translocase (CACT) [57] reduces fatty acid oxidation by impeding their transport from the cytosol to the mitochondria.

Polyunsaturated fatty acids are known to suppress hepatic lipogenesis, whereas saturated and monounsaturated fatty acids have minimal to no effect on fatty acid synthesis [44,58]. Additionally, an argan oil enriched diet prevents the hyperlipidemic effects associated with LPS by inducing the expression of hepatic nuclear receptors, including PPARα, Estrogen-related receptor (ERR) α, and their coactivator PGC-1α, thereby upregulating their mitochondrial and peroxisome target genes involved in fatty acid oxidation [59]. This highlights the beneficial impact of argan oil on lipid metabolism.

## 3. Nrf2

The main redox-sensitive transcription factor, the Nrf2 protein, is a master regulator for oxidative stress management in mammalian cells that maintains cellular homeostasis through controlling the redox balance, xenobiotic metabolism [60], the metabolism of carbohydrates [61], lipids and iron [62], antioxidant and anti-inflammatory responses, protein folding, and proliferation. Nrf2 was first discovered in 1994 [63]. It is transcribed from the human *NFE2L2* gene and belongs to the cap‘n’collar subclass (CNC) of the basic leucine zipper transcription factor protein family (bZIP) along with five other nuclear factors: NF-E2, Nrf1, Nrf3, Bach1, and Bach2 [64]. It has been mapped to the long arm of human chromosome 2 (2q31.2) [65]. Nrf2 is expressed in all tissues, and especially in the organs involved in detoxification processes and metabolism [66,67]. Nrf2 is characterized by an extremely short cellular half-life of approximately 15 to 40 min through degradation by the ubiquitin proteasome arsenal [68]. This short-lived protein is known for its pivotal role in combating harmful ROS formation as well as associated inflammatory and metabolic responses. Nrf2 orchestrates the expression cytoprotective gene encoding phase II detoxifying enzymes [69,70] as well as the expression of genes involved in mitochondrial function and biogenesis [71]. Nrf2 translocates into the nucleus, heterodimerizes with the Maf or Jun protein, and then binds to its antioxidant response elements (AREs) to trigger the transcription of cytoprotective genes [72]. Interestingly, the Nrf2 signaling pathway was found necessary for hematopoietic cell differentiation [6], accelerating cell proliferation, neovascularization, and the repair of damaged tissues [73]. Moreover, it upregulates the expression of antioxidant genes, inhibits microglia-mediated inflammation, and improves mitochondrial function in neurodegenerative diseases [67,74,75]. In addition, it has been reported that Nrf2 expression loss is strongly associated with the metastatic behavior of cancer cells and tumor malignancy [76]. Altogether, Nrf2 is considered to be the pro-survival factor that orchestrates the production of cytoprotective machinery components.

### 3.1. Nrf2 Structure and Function

**Figure 1:**
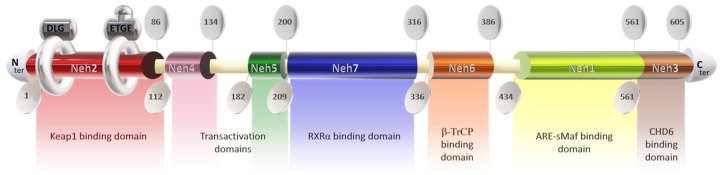
Structure of the protein Nrf2. Keap1: Kelch-like ECH-associated protein 1; Neh domain: N-amino terminal end harbors; RXRα: Retinoic X receptor α; ARE: antioxidant response element; s-Maf: small musculoaponeurotic fibrosarcoma factor; CHD6: Chromodomain helicase DNA binding protein 6.

The Nrf2 protein consists of 605 amino acids and features seven highly conserved functional domains (Neh1-7), notably including the Neh2 domain at the N-terminal, which is responsible for interacting with the negative regulator Keap1 [77]. This domain contains DLG and ETGE motifs, with the latter exhibiting a higher affinity for Keap1 [78,79,80,81,82]. Disruption of the DLG motif suppresses Nrf2 ubiquitination [72,76], while lysine residues between DLG and ETGE motifs are crucial for ubiquitin conjugation and Nrf2 stability [79,83]. Additionally, serine residue ser40 facilitates Nrf2 nuclear translocation post-dissociation from Keap1 [79] (Figure 1).

The Neh4 and Neh5 domains promote the interaction of Nrf2 with coactivators [80], enhancing the transactivation of target genes [84]. Neh5 also regulates Nrf2 cytoplasmic localization [85]. Neh7 serves as a repressive domain inhibiting Nrf2-RXRα binding [86], while Neh6 negatively regulates Nrf2 stability [87]. Neh1 facilitates DNA binding of Nrf2 to antioxidant response elements (ARE/EpRE) [88,89,90], promoting gene expression and downregulating pro-inflammatory mediators. The Neh1 domain contains a leucine zipper for heterodimerization with the small musculoaponeurotic fibrosarcoma factor (sMAF) and a nuclear localization signal for translocation to the nucleus [91,92,93]. The Neh3 domain in the C-terminal region mediates coactivator interactions [80,84].

**Figure 2:**
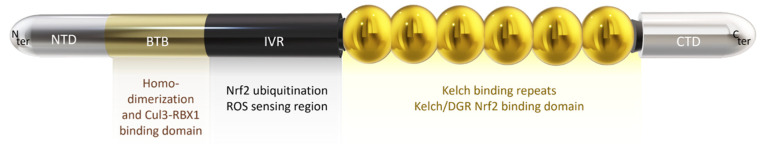
Structure of the protein Keap1. NTD: N-terminal domain; BTB: 2-a broad complex, tram-track, bric-a-brac; IVR: 3-a cysteine-rich intervening region; CTD: C-terminal domain.

Keap1, a dimeric cytoplasmic protein, functions as a major Nrf2 inhibitor (Figure 2). It contains an NTD, a BTB domain for Neh2 interaction, an IVR with an NES for cytoplasmic localization, a DGR with Kelch motifs essential for KEAP1-Nrf2 association, and a CTR [83,94,95,96,97]. Keap1’s structure and function are crucial in regulating the Nrf2 system.

### 3.2. Nrf2-KEAP1 Signaling Pathway

Nrf2 and KEAP1 are crucial for cellular defense mechanisms, maintaining redox homeostasis, and controlling cell fate [98]. In physiological conditions, Nrf2 is ubiquitinated by KEAP1/CUL3–RBX1 and degraded by proteasomes to keep basal gene expression [64,92,99]. KEAP1 cysteine residues (Cys151, Cys273, and Cys288) facilitate rapid Nrf2 degradation, while the ATP-dependent segregase p97 also promotes Nrf2 degradation [100]. Alternatively, proteins like β-TrCP and HRD1 trigger Nrf2 ubiquitination and degradation [101]. The oxidation of KEAP1 cysteines in noncanonical pathways leads to Nrf2 release [101]. Nrf2 contains redox-sensitive cysteines, preventing KEAP1 binding [102,103,104]. Several regulators, including p62, WTX, and DPP3, stimulate Nrf2 by binding KEAP1 [104,105,106]. Upon activation, Nrf2 translocates to the nucleus, heterodimerizes with sMaf proteins, and binds to ARE sequences, inducing cytoprotective gene expression [107,108]. Nrf2 regulates numerous genes related to vital cellular functions [109,110]. After activation, Nrf2 is phosphorylated, leading to nuclear export and degradation [70,77,111,112,113]. Interaction with RXRα inhibits Nrf2 activity [114]. Transcriptional and post-transcriptional regulation significantly affects Nrf2 activity [115]. 

### 3.3. Nrf2 and Oxidative Stress 

Mammalian cells are regularly threatened by multiple stress sources within their immediate microenvironment. Thence, they are commonly armed with a strong arsenal of non-enzymatic compounds, such as glutathione (GSH), vitamin C (ascorbate), and vitamin E (tocopherols) [116,117,118], and antioxidant enzymes (SODs, catalase, thioredoxins, peroxiredoxins, and glutathione peroxidases) [119,120], participating in the adaptive regulatory mechanisms to maintain the cellular and tissue homeostasis [79]. The presence of oxidant molecules such as ROS and reactive nitrogen species (RNS) can generate an imbalance in the redox status, which usually causes damage to cellular macromolecules (lipids, proteins, and DNA). The consequences of this oxidative stress are cell death and the development of metabolic and chronic diseases, including atherosclerosis, autoimmune disorders, diabetes, osteoporosis, rheumatoid arthritis, and neurodegenerative diseases (Parkinson’s disease and Alzheimer’s disease), among others [68,121]. In 1985, Sies defined oxidative stress (OS) as “*a disturbance in prooxidant-antioxidant balance in favor of the former*” [122]. Dean Jones added to this definition that OS is also the disruption of redox signaling circuitries [123]. In addition to their cytotoxicity, ROS have an important role in vital cellular functions. At limited concentrations, they play a major role in proliferation, differentiation, inflammation, immune function, autophagy, and stress response by acting as a second messenger [124]. However, an elevated concentration of ROS may activate oncogenes, inactivate tumor suppressor genes, and promote mitochondrial malfunction [125]. As the redox master chief regulator, Nrf2 is activated by a plethora of ARE inducers, known as stressors, including oxidative and chemical stress provoked by hydrogen peroxide (H_2_O_2_) [126], NO [127], tertiary butylhydroquinone (tBHQ) [128], and fumarate (DMF) [129]. Amid the natural phytochemicals, silymarin, sulforaphane, curcumin, cinnamic aldehyde [130], and bardoxolone methyl are the most well studied. Numerous studies have reported the efficient effect of Nrf2 activation on the expression and production of several antioxidant enzymes to maintain the oxidative balance in eukaryotic cells (Table 4). In diabetic mice, the Nrf2/KEAP1 signaling pathway protects pancreatic β-cells by weakening oxidative damage through the induction of its antioxidant enzymatic system, which inhibits apoptosis and proliferation [131]. Accumulating evidence identifies the crosstalk between Nrf2 and NF-κB signaling pathways to maintain redox balance and to regulate the cellular response to stress and inflammation [132]. In age-related macular degeneration model, The H_2_O_2_ induced levels of ROS and MDA, while the Nrf2-related antioxidant enzymes SOD, GPx, and the reduced glutathione (GSH) were upregulated. These results were explained by the fact that Nrf2 induces the expression of heme oxygenase-1 (HO-1), a potent antioxidant enzyme [133]. Similarly, Jung and al. reported that Nrf2 activation increases the cellular HO-1 level, subsequently impeding the degradation of IκB-α [134], which inhibits NF-κB nuclear translocation [134,135]. Therefore, Nrf2 suppress the expression of NF-κB pro-inflammatory target genes. In addition, the administration of α-mangostin, an Nrf2 activator, enhanced antioxidant cell defense and reduced pro-inflammatory cytokines, including tumor necrosis factor (TNF-α) and interleukin-1β and -6 (IL-1β, IL-6), and contributed to the restoration of hepatic GSH, SOD, and catalase activities [136].

**Table 4:** Nrf2 target genes and biochemical functions.

Enzyme Name	Protein Symbol	Biochemical Function
**Alcohol dehydrogenase**	ADH	Antioxidant and detoxification enzymes [137,138]
**Aldehyde dehydrogenase**	ALDH	
**Aldo-keto reductase family 1**	AKR1	
**ATP-binding cassette subfamily B/C**	ABCB/ABCC	
**Carbonyl reductase**	CBR	
**Catalase**	CAT	
**Cytochrome P450**	CYP1B1	
**Epoxide hydrolase, microsomal**	EXPH	
**Glutamate-cysteine ligase**	GCL	
**Glutathione peroxidase**	GPx	
**Glutathione reductase**	GR	
**Glutathione S-transferase**	GST	
**Glutathione synthase**	GSS	
**Heme oxygenase-1**	HO-1	
**NADPH-quinone oxidoreductase-1**	NQO-1	
**Peroxiredoxins**	PRDX1	
**Prostaglandin reductase**	PTGR	
**Sodium independent cysteine glutamate antiporter**	SLC7A11	
**Sulfiredoxin1**	SRXN1	
**Superoxide dismutase**	SOD	
**Thioredoxin**	TXN	
**Thioredoxin reductase**	TXNRD	
**UDP-glucuronosyl transferase**	UGT	
**Ferritin light/heavy chain**	FTL /FTH	Iron metabolism [139]
**Glucose-6-phosphate dehydrogenase**	G6PD	Bioenergetic function [140]
**6-phophogluconate dehydrogenase**	PGD	
**Malic enzyme**	ME	
**NADP-dependent isocitrate dehydrogenase**	IDH	
**B-cell lymphoma**	BCL	Apoptosis [141]
**Autophagy protein**	ATG	Autophagy [141]
**Microtubule-associated protein1A/1B-light chain 3B**	LC3B	
**Activating transcription factor**	ATF	Proteasomal degradation [142]
**Proteasomes**	PSM	
**Sequestosome (p62)**	SQSTM	

## 4. Nrf2 and Argan Oil Compounds

### 4.1. Nrf2 and Oleic and Linoleic Acids

Oleic acid (C18:1n-9) and linoleic acid (C18:2n-6) are common unsaturated fatty acids present in various dietary fat sources [143]. Argan oil contains a balanced ratio of oleic acid (46.3%) and linoleic acid (34%), whereas olive oil is predominantly composed of oleic acid (73%), with a lower linoleic acid content (11.3%). The oxidation of linoleic acid produces metabolites with conflicting roles as both beneficial and detrimental components [144,145]. One such product is 12,13-epoxy-9-keto-10(trans)-octadecenoic acid (EKODE), which activates ARE in primary cells and IMR-32 human neuroblastoma cells, mediated by the transcription factor Nrf2 [146]. A novel fatty acid metabolite, 10-oxo-trans-11-octadecenoic acid, generated by gut *Lactobacillus plantarum*, increases Nrf2 levels and induces antioxidant enzyme expression in mouse tissues and HepG2 cells [147].

Olive oil and its component hydroxytyrosol elevate Nrf2 activation and protein expression in fibroblasts, while oleic acid increases ROS production [148,149]. Roselle seed oil, rich in linolenic (30%), palmitic (25,8%), and oleic acids (14.45%), enhances Nrf2 levels in rat livers exposed to paracetamol [150]. Oleic acid treatment increases Nrf2 expression in HepG2 cells, but it does not affect Nrf2 in steatosis-induced HL-7702 cells [151,152]. In a rabbit model of acute respiratory distress syndrome, oleic acid does not alter Nrf2 levels [153]. However, oleic acid has been found to protect hepatic cells from H_2_O_2_-induced inflammation and oxidative stress. Accordingly, supplementation with oleic acid upregulates Nrf2 mRNA expression, contributing to its protective effects against hepatic ischemia-reperfusion injury in mice, possibly through the inhibition of AKT/mTOR pathways [154].

### 4.2. Nrf2 and Ferulic Acid

Ferulic acid, a common phenolic compound, is abundantly present in numerous fruits, vegetables, and vegetable oils such as argan oil [155] and olive oil [156] (Table 5). 

Recently, several derivatives of ferulic acid with enhanced activity and improved stability and toxicity have been identified, making them promising candidates for various applications. Ferulic acid itself has demonstrated effectiveness in managing apoptosis, fibrosis, inflammation, platelet aggregation, oxidative stress, and vascular endothelial injury [157]. Studies consistently report the modulation of Nrf2 by ferulic acid at both mRNA and protein levels, although specific mechanisms may vary across cell models. For instance, in IPEC-J2 porcine enterocytes pretreated with deoxynivalenol, ferulic acid exposure decreased cytoplasmic Nrf2 content while increasing its nuclear translocation, accompanied by reduced Keap1 expression and elevated HO-1 expression, indicating Nrf2 pathway activation to mitigate oxidative stress [158]. Likewise, in irradiated human umbilical vein endothelial cells, ferulic acid facilitated nuclear Nrf2 translocation via extracellular signal-regulated kinase and phosphatidylinositol 3-kinase pathways, offering protection against radiation-induced oxidative stress [159].

An increased nuclear accumulation of Nrf2 was observed in hepatocytes treated with *Nelumbo nucifera* leaves, containing various phenolic compounds including ferulic acid, suggesting Nrf2-mediated upregulation of antioxidant enzymes (i.e., CAT, HO-1, and SOD-1) [160]. Ferulic acid also exhibited neuroprotective effects in SH-SY5Y neuroblastoma cells by inducing HO-1 expression and promoting Nrf2 nuclear translocation [161]. In PC12 cells (derived from rat pheochromocytoma) exposed to lead acetate, ferulic acid induced HO-1 gene expression and enhanced ARE promoter activity, indicating its potential for treating lead neurotoxicity in children [162]. In rat livers, the administration of ferulic acid increased the total Nrf2 protein expression and antioxidant gene expression, suggesting Nrf2 accumulation and ARE activation post-treatment [163,164]. In addition, the administration of mice with the ethyl acetate extract of purple rice was found to enhance the activities of GPx in mice livers and sera concomitantly to the increase in the Nrf2 expression. The characterization of this extract by ultra-high-performance liquid chromatography tandem mass spectrometry determined quercetin and ferulic acid as the primary phenolic compounds [165]. Moreover, the effect of the administration of ferulic acid was investigated against retinal photooxidative damage in pigmented rabbits. The HO-1 mRNA and protein levels were increased by ferulic acid after light exposure. In line with these modifications, the treatment with this phenolic compound reinforced the light-induced increase in both the Nrf2 protein and Nrf2 mRNA levels [166].

Ferulic acid treatment of carp resulted in a reduction in ROS formation caused by difenoconazole and restored CAT activity and successfully repressed the transcript levels of genes involved in the Nrf2 signaling pathway, including Keap 1, Nrf2, and HO-1. Consequently, FA restored the Nrf2 signaling pathway, thereby improving spleen function and reducing oxidative stress [167]. In another study investigating the effect of ferulic acid against methoxyethanol-induced testicular oxidative stress in rats, it was observed that ferulic acid increased the activities of GPx, CAT, and SOD in the testicles. By contrast, it significantly decreased the MDA level and Nrf2 expression [168]. Collectively, these studies provide diverse perspectives on the Keap1/Nrf2/ARE signaling pathway regulated by ferulic acid. While some studies support its activation, others contradict these findings, highlighting the need for further research to clarify the underlying mechanisms and factors governing ferulic acid’s interaction with the Nrf2 pathway. 

In the case of increased antioxidant enzyme levels under oxidative stress, there are distinct scenarios. In one scenario, ROS oxidize the main cysteine residues of Keap1, triggering conformational changes that prevent Nrf2 binding and its transfer to the nucleus and subsequent activation of ARE-mediated gene expression, including GPx, CAT, and SOD. Another scenario occurs when the ROS attack exceeds the cellular defense capacity, resulting in the inadequate production of protective enzymes. Alternatively, when ROS attack and defense levels are balanced, cells increase the production of protective enzymes, leading to increased antioxidant enzyme levels. Subsequently, as oxidative stress diminishes due to ROS scavenging, the negative feedback mechanism reduces Nrf2 synthesis, resulting in a decline in antioxidant enzyme levels below the limit of detection. 

### 4.3. Nrf2 and Phytosterols (Schottenol and Spinasterol)

**Figure 3:**
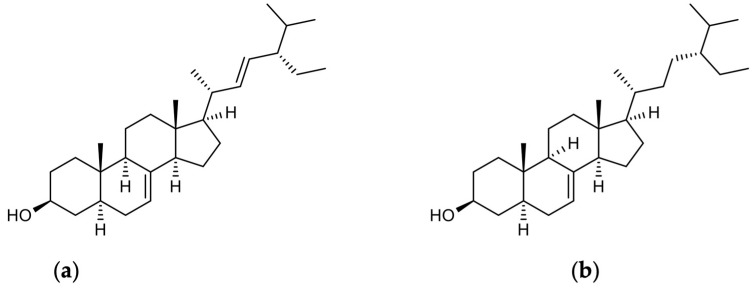
Chemical structures of spinasterol and schottenol: (**a**) alpha-spinasterol; (**b**) schottenol.

In the last few years, much attention has been given to phytosterol-enriched foods [169]. Phytosterols are structurally related to cholesterol and are mainly C-28 and C-29 carbon steroid alcohols (Table 5) [170]. Schottenol and spinasterol (Figure 3) are two phytosterols mainly present in argan oil [15] and less in cactus seed oil [171]. To the best of our knowledge, there have been no studies investigating the direct impact of spinasterol or schottenol on the Nrf2 signaling pathway. However, we have recently explored how oxidative stress is influenced by these two phytosterols. Given that Nrf2 plays a crucial role as a transcription factor in cellular defense against oxidative stress, a potential association between the Nrf2 signaling pathway and both phytosterols could be suggested. Specifically, schottenol has been found to increase mitochondrial membrane potential, indicating mitochondrial hyperpolarization in BV2 cells [172] Additionally, phytosterol did not affect the cell growth of SK-N-BE human neuronal cells [17]. Furthermore, both schottenol and spinasterol reduced intracellular ROS and NO levels in the culture medium. Conversely, they enhanced ACOX1 activity in microglial BV-2 cells and normalized CAT activity in both wild-type and ACOX1-deficient microglial cells. These findings collectively suggest the potential of schottenol and spinasterol to provide protection against oxidative stress [16]. Another oil derived from cactus seed demonstrated hepatoprotective and neuroprotective effects against LPS-induced damage by restoring peroxisomal antioxidant and β-oxidative capacities in mouse livers, thereby maintaining brain peroxisomal antioxidant activities, including glutathione peroxidase and catalase [171].

### 4.4. Nrf2 and Tocopherols

Tocopherols (Table 5) available in nuts, vegetable oils, and some oilseeds, come in four lipid-soluble forms, α, β, γ, and δ, each with a distinct arrangement of methyl substituents on the chromanol ring and hydrocarbon side chain [173]. 

The antioxidant capacity of tocopherol isomers depends on the of hydroxyl groups and follows the order of α > β > γ > δ [174]. However, γ- and δ-tocopherols possess unique antioxidant properties not seen in α-tocopherol [175]. Argan oil contains mainly γ- and δ-tocopherol, with 81–92 g/100 g and 6.2–12.8 g/100g, respectively [176]. Interestingly, the unsaponifiable fraction of roselle seed oil, rich in γ-tocopherol (150 mg/100 g), α-tocopherol (58.7 mg/100 g), and δ-tocopherol (27.16 mg/100 g), raises the hepatic Nrf2 level in rats treated with paracetamol, concurrently reducing MDA levels and increasing glutathione (GSH) [150]. γ-Tocopherol treatment (25 µM) recovered cell viability in Hepa1c1c7 cells subjected to hydrogen peroxide [177]. Moreover, *Rosa rubiginosa* L., rich in α- and γ-tocopherols, was found to increase the hepatic Nrf2 level and increased its ARE binding capacity in rats subjected to ischemia followed by reperfusion. In addition, the mRNA expression of HO-1 was increased [178]. However, the elimination of α- and γ-tocopherols from *Rosa rubiginosa* L. prevented its protective effect against high-fat-diet induced Nrf2 depletion in the livers of mice, contrasting with the beneficial effects observed when both tocopherols were present [179]. In addition, it has been shown that γ-tocopherol decreased MDA levels without changing Nrf2 expression in mice [180]. Exposure of Human Retinal Pigment Epithelium to α-tocopherol followed by tert-butyl hydroperoxide increased Nrf2 expression 3.5-fold, while γ-tocopherol had no effect [181]. In rats, an enriched diet with γ-tocopherol mixtures increased liver Nrf2 protein levels while maintaining Keap1 levels. These findings collectively suggest that tocopherols modulate the Nrf2 signaling pathway differently depending on the dosage and cell model [182]. 

**Table 5:** Biological activities of major argan oil compounds as Nrf2 pathway regulators in preclinical studies.

	Compound/Structure	Effective Concentration	Biological Activities	Nrf2 Downstream Genes	Disease(s)	Study Model	References
**1**	Linoleic acid	1–10 μM	Antioxidant	NQO1	Oxidative stress	IMR-32 neuroblastoma cells and cerebro-cortical neurons	[146]
		˃1 µM	Antioxidant	NQO1, HO-1, and GCLM	Oxidative stress	HepG2 cells	[147]
		˃1 µM	Antioxidant	HO-1	Oxidative stress	Murine dermal fibroblast	[148]
		0.6, 4 and 8 mL/kg	Antioxidant; anti-inflammatory	HO-1	Inflammation	Rats	[150]
		1 mM	Antioxidant	HO-1	Oxidative stress	Mice	[151]
**2**	Oleic acid	100 µM	Antioxidant; anti-inflammatory	-	Hepatic ischemia-reperfusion (I/R) injury	Mice	[154]
**3**	Ferulic acid	0–100 μM	Antioxidant; anti-inflammatory	SOD, CAT	Oxidative stress, inflammation, and apoptosis	IPEC-J2 cells	[158]
		0.2–5 µM	Antioxidant	NQO1, HO-1, GCLM, and GCLC	Oxidative stress	Human umbilical vein endothelial cells (HUVECs)	[159]
		50 mg/kg	Antioxidant	NQO1, HO-1, GST, SOD, CAT, and GPx	Oxidative stress	Rats	[168]
**4**	Alpha-tocopherol	0.01 mL/g	Antioxidant; anti-inflammatory	HO-1	Oxidative stress and obesity	Mice	[179]
		100 μM	Antioxidant	SOD	Oxidative stress	hTERT-RPE cells	[181]

## 5. Conclusions

When compared to other edible oils, Argan oil shows an unique composition, with high levels of linoleic and oleic acids, as well as polyphenols, sterols, and tocopherols. Nrf2 is the main redox-sensitive transcription factor that regulates the expression of genes that encode antioxidant molecules. This pathway plays a crucial role in controlling the cellular antioxidant response against oxidative damage. Major argan oil constituents have been shown to regulate the Nrf2 pathway, and their antioxidant properties highlight the natural resource’s possible therapeutic uses in enhancing cellular defense systems and reducing oxidative stress. This pathway plays a crucial role in controlling the cellular antioxidant response against oxidative damage. The antioxidant qualities of argan oil are attributed to its constituents, including fatty acids, polyphenols, and tocopherols. Such structurally diverse chemicals can scavenge free radicals, prevent lipid peroxidation, and enhance cellular resilience. These findings underline the chemical basis that gives argan oil its antioxidant properties and highlight its promising medicinal uses. Of note, minor components of argan oil, such as alpha-linolenic acid (ALA), contribute to its nutritional profile and can activate the skn-1/Nrf2 transcription factor, offering potential benefits at different levels by inhibiting oxidative stress, promoting longevity, and exerting anti-inflammatory effects. Moreover, the activation of the Nrf2 signaling pathway by gamma-linolenic acid (GLA) can alleviate muscle atrophy and increase antioxidant defense mechanisms. Collectively, this research highlights the interesting interplay between natural compounds and cellular defense systems, making the natural argan oil a sustainable option for improving health by curing oxidative-stress-related diseases. Future studies will further explore the dietetic potential of argan oil, paving the way for comprehensive healthcare approaches.

## Figures and Tables

**Table 1:** Comparison of fatty acid composition of argan oil with other natural oils.

Fatty Acids (%)	C16:0 (Palmitic Acid)	C18:0 (Stearic Acid)	C18:1 (Oleic Acid)	C18:2n-6 (Linoleic Acid)	References
Oils					
**Argan oil**	12.72	5.45	45.97	34.75	[23]
**Olive oil**	9.7	2.6	73.6	11.3	[12]
**Sunflower oil**	2.54	1.96	16.33	76.96	[24]
**Soybean oil**	Not determined	3.04	24.02	40.00	[25]
**Almond oil**	6.4	0.5	71.1	19.5	[26]
**Peanut oil**	8.77–12.03	3.20–4.38	39.14–56.99	27.07–42.94	[27]
**Corn oil**	12.94	2.12	31.97	48.97	[28]
**Grape seed oil**	7.2	4.8	19.9	68.1	[29]
**Sesame oil**	9.7	6.5	41.5	40.9	[30]

**Table 2:** Comparison of polyphenol composition of argan oil with other natural oils.

	Polyphenol Composition (µg/kg of Oil)				References
	Vanillic Acid	Syringic Acid	Ferulic Acid	Tyrosol	
**Argan oil**	67	37	3147	12	[20]
**Olive oil**	359	ND	51	19,573	[20]
**Corn oil**	4540	910	7830	ND	[35]
**Sesame oil**	ND	1850	ND	930	[36]

ND: Not Determined.

## Data Availability

Data are contained within the article.
